# Thermodynamics and Catalytic Properties of Two Novel Energetic Complexes Based on 3-Amino-1,2,4-triazole-5-carboxylic Acid

**DOI:** 10.1021/acsomega.1c06052

**Published:** 2022-01-11

**Authors:** Huan Song, Bing Li, Xuezhi Gao, Fenglin Shan, Xiaoxia Ma, Xiaoyan Tian, Xiaoyan Chen

**Affiliations:** † State Key Laboratory of High-efficiency Utilization of Coal and Green Chemical Engineering, Ningxia University, Yinchuan 750021, P. R. China; ‡ Department of Chemistry & Chemical Engineering, Ningxia University, Yinchuan 750021, P. R. China

## Abstract

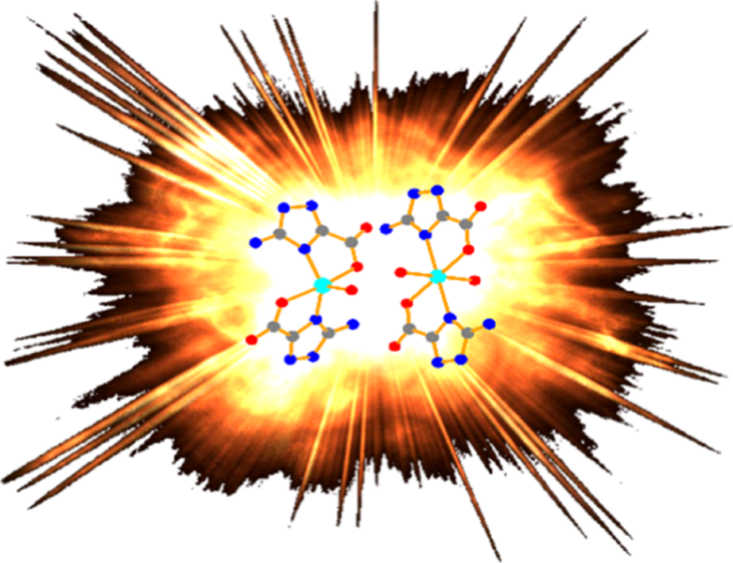

For energetic materials (EMs), the key point of the present research is to improve the energetic property and reduce sensitivity. In this work, two new energetic complexes, Mn(atzc)_2_(H_2_O)_2_·2H_2_O (**1**) and Zn(atzc)_2_(H_2_O) (**2**) (Hatzc = 3-amino-1,2,4-triazole-5-carboxylic acid), were synthesized by solvent evaporation and diffusion methods, respectively. The structural analyses illustrate that **1** and **2** exhibit zero-dimensional structural units, which are linked by hydrogen-bonding interactions to give three-dimensional supramolecular architectures. For complexes **1** and **2**, the detonation velocities (D) are 10.4 and 10.2 km·s^–1^ and detonation pressures (*P*) are 48.7 and 48.6 GPa, respectively. They are higher than most of the reported EMs, which present prominent detonation characteristics. In addition, two complexes can accelerate the thermal decomposition of ammonium perchlorate and exhibit excellent catalytic activity. Therefore, the two complexes can serve as a new class of promising EMs, which have potential application in the design of new high-efficiency solid catalysts.

## Introduction

Energetic materials (EMs) are one of the most important components of organics with an irreplaceable role in solid propellants, which possess special properties of energy storage and stability.^1−3^ However, the currently used EMs, such as hexanitrohexaazaisowurtzitane (CL-20),^4^ 1,3,5-triamino-2,4,6-trinitrobenzene (TATB),^5^ and 1,3,5,7-tetranitro-1,3,5,7-tetrazocine (HMX),^6^ have some limitations due to their high sensitivity or relatively low catalytic activity. Therefore, it is challenging to design and synthesize EMs with high catalytic activity, high heat of detonation, low sensitivities, and environmental acceptability.^7−9^ Recently, nitrogen-rich organic materials have attracted immense attention because they produce eco-friendly N_2_ gas and release enormous energy during the process of decomposition.^10−12^ Nevertheless, the conflict of high energy and oxygen balance cannot be resolved completely. The valid strategy is to construct stable ligands containing poly-nitrogen and oxygen-rich fragments.^13,14^ 3-Amino-1,2,4-triazole-5-carboxylic acid (Hatzc) is one of the high energetic ligands, which possesses a high nitrogen content (N % = 43.8) and possesses high enthalpy of formation from the powerful energy release of C–N, N–N, and N=N bonds.^15,16^ What is more, Hatzc also presents O atoms from carboxylic groups, which can provide a sufficient oxygen content during the explosion.^17^

Ammonium perchlorate (AP) is a commonly used oxidant, which is widely used as the main component of solid rocket propellants.^18^ The thermal decomposition performance of AP can affect the combustion behavior of solid propellants directly.^19^ In the past, the combustion catalysts in propellants were mainly composed of metal oxides, inorganic salts, and organic salts,^20,21^ and these materials were mostly inert catalysts which do not provide energy and even lose a part of the heat, reduce the performance of the propellant during the combustion process.^22^ However, the energetic complexes can provide a diverse structure and greater heat of formation. What is more, they can also provide relatively high heat and fresh metal oxides on the propellant surface, which may improve combustion performance.^23^ Therefore, the energetic complexes can be applied as an additive to the combustion catalytic of propellants.

Based on the above considerations, two energetic nitrogen-rich metal complexes, Mn(atzc)_2_(H_2_O)_2_·2H_2_O (**1**) and Zn(atzc)_2_(H_2_O) (**2**), were designed and synthesized by solvent evaporation and diffusion methods, respectively. Both complexes have excellent detonation performance and low sensitivity. The catalytic performances show that complexes **1** and **2** can accelerate the decomposition of AP.

## Results and Discussion

### Crystal Structure

**Figure 1:**
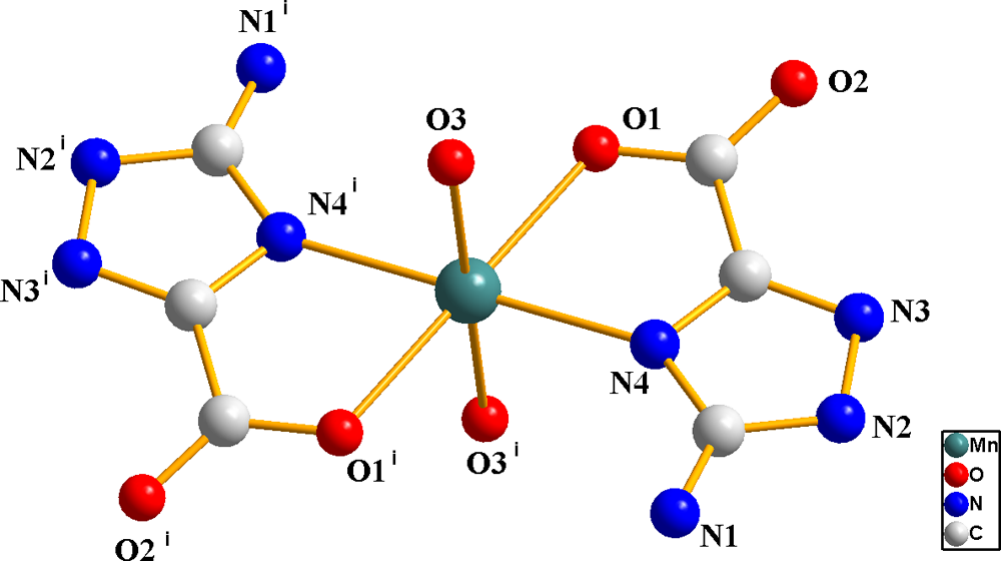
Coordination environment of complex **1** (hydrogen atoms are omitted for clarity).

**Figure 2:**
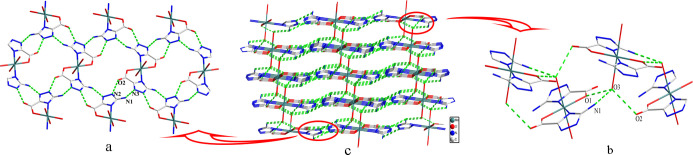
(a) Hydrogen bond modes leading to a 2D supermolecular layer. (b) Hydrogen bond modes leading to a 3D structure. (c) 3D supermolecular network of 1.

Crystal structure of Mn(atzc)_2_(H_2_O)_2_·2H_2_O. Complex **1** crystallizes in triclinic space group *P*1̅ (Table S1). The asymmetric unit of **1** comprises one crystallographically independent Mn(II) ion, two atzc^–^, two coordinated water molecules, and two free water molecules (Figure 1). Each Mn(II) ion displays a slightly distorted octahedral geometry. The equatorial plan is defined by two oxygen atoms (O1 and O1^i^) and two nitrogen atoms (N4 and N4^i^) from two Hatzc ligands, and the axial position is occupied by two oxygen atoms (O3 and O3^i^) from two coordination water molecules. The bond length of Mn1–O3 [2.209(3) Å] is slightly shorter than that of Mn1–O1 [2.246(3) Å] (Table S2). Furthermore, due to the presence of abundant hydrogen bonds (Table S3), N(2)–H(2)···O(2) and N(1)–H(1B)···N(3) hydrogen bonds, the zero-dimensional motif is linked into two-dimensional (2D) supramolecular layers (Figure 2a). Then, these layers are cross-linked by O(3)–H(3A)···O(1), O(3)–H(3B)···O(2), and N(1)–H(1A)···O(3) (Figure 2b) to create a three-dimensional (3D) supramolecular architecture (Figure 2c).

**Figure 3:**
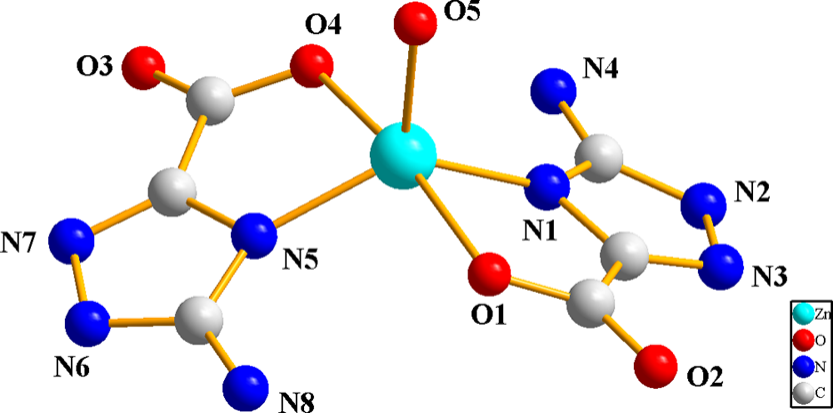
Coordination environment of complex **2** (hydrogen atoms are omitted for clarity).

**Figure 4:**
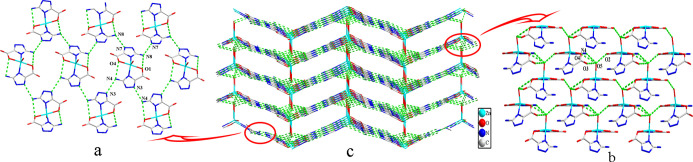
(a) Hydrogen bond modes leading to a 2D supermolecular layer. (b) Hydrogen bond modes leading to a 3D structure. (c) 3D supermolecular network of **2**.

Crystal structure of Zn(atzc)_2_(H_2_O) **(2)**. Single-crystal structure analysis reveals that complex **2** crystallizes in monoclinic space group *P*2_1_/*c* (Table S1). The asymmetric unit is consisted of a Zn(II) ion, two atzc^–^, and one coordinated H_2_O molecule. As shown in Figure 3, the Zn ion is five-coordinated by three O atoms (O1, O4, and O5) and two N atoms (N1 and N5), forming a distorted tetragonal pyramidal geometry. Among them, two N atoms (N1 and N5) and two O atoms (O1 and O4) are coplanar, and Zn1–O5 is at the axial site with an O5–Zn1–O1 bond angle of 93.5(2)°. The Zn–N and Zn–O bond lengths vary from 1.961(8) to 1.986(7) Å and 1.971(5) to 2.167(5) Å, respectively (Table S2). The distorted tetragonal pyramidal motifs can be linked into 2D supramolecular layers (Figure 4a) *via* the intramolecular hydrogen bonds N(8)–H(8B)···O(1) and N(4)–H(4B)···O(4) and the intermolecular hydrogen bonds N(8)–H(8A)···N(7), N(7)–H(7)···N(8), N(4)–H(4A)···N(3), and N(3)–H(3)···N(4). Then, a 3D supramolecular structure is generated through a number of hydrogen bonds (Table S3) N(4)–H(4B)···O(5), O(5)–H(5B)···O(2), O(5)–H(5A)···O(3), and O(5)–H(5A)···O(4) (Figure 4b) between atzc^–^ and the coordinated H_2_O molecule (Figure 4c).

### Thermal Stability

The thermal decomposition of the complexes was studied by thermogravimetric (TG) experiments, which are important parameters for EMs. The TG curve suggests that **1** and **2** undergo two weight-loss stages (Figure S1). They begin to decompose at 120 and 140 °C with endothermic peaks at 225 and 215 °C, which correspond to the expulsion of coordinated water molecules and free water molecules. The 18.4% weight loss of complex **1** at 120–240 °C is attributed to the release of water molecules (calcd: 18.9%). Complex **2** losses coordinated water molecules in the temperature range of 195–251 °C with a weight loss of 4.9% (calcd: 5.3%). Then, the main framework collapses with exothermic peaks at 425 and 515 °C, respectively. **1** and **2** completely convert to MnO_2_ and ZnO with residue weights of 24.1 and 24.3%, which is in agreement with the calculated values of 25.2 and 24.5%, respectively.

### Energetic Properties

The enthalpies of formation (Δ_f_*H*^o^) of the two complexes were calculated by Hess thermochemical cycle and deduced as 0.21 and 0.56 MJ·kg^–1^, respectively. To confirm that detonation velocity (*D*) and detonation pressure (*P*) of detonation characteristic for EMs were estimated based on the EXPLO5 code (Table S4), they were usually applied to the energetic metal–organic frameworks reported previously.^25^ For complexes **1** and **2**, the *D* values are 10.4 and 9.9 km·s^–1^ and the *P* values are 48.7 and 47.5 GPa, respectively. They are higher than most of the reported EMs. The sensitivity test examined the impact and friction sensitivities of **1** and **2** (Table 1) to reflect the safety of EMs. The impact sensitivity values of **1** and **2** are all greater than 40 J, and the friction sensitivity values of **1** and **2** are higher than 360 N, which are “insensitive”.^26^ Complexes **1** and **2** have low sensitivity owing to the presence of a large number of hydrogen bonds in the structures.

**Table 1:** Physicochemical Properties of **1** and **2** and Relevant Energetic Complexes

compound	ρ^a^ (g·cm^–3^)	*N*^b^ (%)	Ω^c^	*T*_dec_^d^ (oC)	*D*^e^ (km·s^–1^)	*P*^f^ (GPa)	IS^g^ (J)	FS^h^ (N)
1	1.853	32.47	–60.26	120	10.4	48.7	>40	>360
2	1.998	33.20	–56.87	140	10.2	48.6	>40	>360
Zn–L^16^	2.108	46.12	–63.25	229	9.6	44.6	>40	>360
Mn–L^16^	1.968	47.75	–70.96	234	9.1	38.2	>40	>360
TNT^28^	1.654	18.50	–74.00	244	7.2	20.5	15	353
RDX^28^	1.806	37.80	–21.60	210	8.6	33.9	7.5	120
CHP^24^	1.948	14.71	–11.48	194	8.2	31.7	0.5	
NHP^24^	1.983	33.49	–11.48	220	9.2	39.7		

a Density from pycnometer.

b Nitrogen content.

c Oxygen balance.

d The onset decomposition temperature (DSC).

e *D*.

f *P*.

g Impact sensitivity.

h Friction sensitivity. Zn–L and Mn–L represent [Zn(Hdtim) (H_2_O)_2_]_4_ and [Mn(Hdtim) (H_2_O)_2_]_4_, respectively.

For complexes **1** and **2**, they exhibit excellent energetic properties and low sensitivity, which are mainly attributed to the formation of hydrogen bonds between intermolecules and intramolecules.^27^

### Effects on the Thermal Decomposition of AP

**Figure 5:**
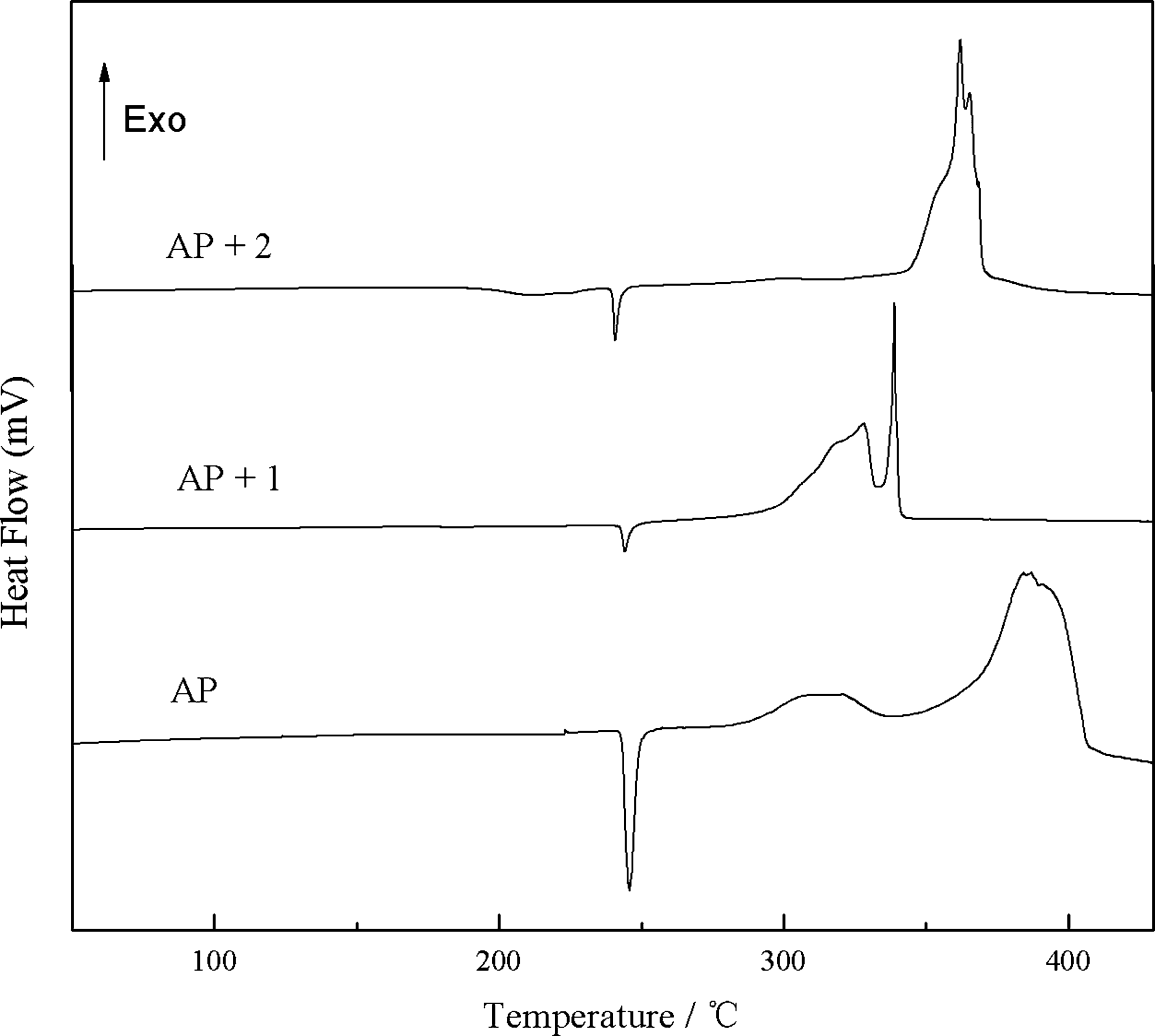
DSC curves for AP, **1** + AP, and **2** + AP at a heating rate of 10 °C·min^–1^.

In order to examine the effect of complexes **1** and **2** on the thermal decomposition of AP, the target sample was prepared by mixing AP and the title complexes at a mass ratio of 1:3. The study was carried out by differential scanning calorimetry (DSC) at 30–450 °C·min^–1^ in a hydrostatic air atmosphere with Al_2_O_3_ as a reference at a heating rate of 10 °C·min^–1^. The study was investigated using DSC measurement at a heating rate of 10 °C·min^–1^ in a hydrostatic air atmosphere in the range of 30–450 °C with Al_2_O_3_ as a reference. The activation energy (*E*_a_) and pre-exponential factor (*A*) of thermal decomposition for AP and AP with complexes were measured at four different heat rates of 5, 10, 15, and 20 °C·min^–1^ by the Kissinger’s method^29^ (Figures S2–S4). Figure 5 shows the DSC curves of AP, AP with **1**, and AP with **2**, respectively. The endothermic peak of pure AP at 245 °C is formed by the phase transformation. The exothermic peaks at 290 and 442 °C are corresponding to the low-temperature decomposition process and high-temperature decomposition process. The heat releases of the exothermic process are 0.735 and 0.787 kJ·g^–1^, respectively. After adding the mixture of AP with **1** and AP with **2**, there are no obvious effects on the phase transition of AP, but the exothermic phase has significant change. For AP with **1**, the exothermic process at 250–450 °C for pure AP becomes narrowed, which appears in the region 255–345 °C. For AP with **2**, the two exothermic peaks combined into one at 342–370 °C. This indicates that the decomposition time of AP is shorter in the presence of complexes at the same heating rate. What is more, the decomposition heat changes to 1.916 kJ·g^–1^ for **1** and 1.568 kJ·g^–1^ for **2**, significantly higher than the corresponding heat value for pure AP. Clearly, AP decomposes completely in a relatively short time and releases a lot of heat in the presence of the title complexes. It can be inferred that the main skeleton of the ligand releases a large amount of heat during the decomposition process, and the formation of metal oxides at the molecular level on the propellant surface may contribute to their catalytic effects.^28^

As shown in Table 2, thermal decomposition peak temperature, activation energy (*E*_a_), and pre-exponent (*A*) were measured by DSC for AP (Figure S2) and AP with **1** (Figure S3) and **2** (Figure S4) at different heat rates. The increases in activation energy and pre-exponential factor are due to the kinetic compensation effect. The ratio of *E*_a_ to ln *A* can be used to describe the reactivity.^30^ Generally, a larger ratio means a greater stability of the reactant. The *E*_a_/ln *A* values of AP with **1** and AP with **2** are 13.56 and 14.01, respectively, which are smaller than those of pure AP. Both complexes serve good acceleration effects toward the thermal decomposition of AP, and the catalytic effect of **1** is better than that of **2**.

**Table 2:** Kinetic Parameters of Thermal Decomposition for AP and AP with Additives

materials	heating rates/^o^C·min^–^^1^	peak temperature/^o^C	*E*_a_/KJ·mol^–^^1^	ln *A*/s^–^^1^	*E*_a_/ln *A*
AP	5	370.9	148.97	9.62	15.49
	10	393.2			
	15	396.9			
	20	397.7			
1 + AP	5	315.4	156.72	11.56	13.56
	10	328.1			
	15	335.6			
	20	340.1			
2 + AP	5	350.7	187.09	13.35	14.01
	10	362.4			
	15	369.7			
	20	374.0			

Compared with other AP decomposition catalyst candidate EMs,^18,23^ these complexes should meet the following requirements. First, the high-energy ligands in these complexes can increase the decomposition heat and favor the thermal decomposition of AP; second, the formation of metals and oxides at the molecular level on the propellant surface during compound decomposition may contribute to the catalytic effect of the catalyst.

## Conclusions

In summary, two new energetic complexes, Mn(atzc)_2_(H_2_O)_2_·2H_2_O (**1**) and Zn(atzc)_2_(H_2_O) (**2**), were synthesized by solvent evaporation and diffusion methods, respectively. Both **1** and **2** exhibit excellent powerful detonation performances and low sensitivities, which make these new complexes as potential EMs. The superior detonation properties of the two energetic complexes are beneficial to the accelerated activity toward the thermal decomposition of AP, which are expected to be candidates for solid catalysts.

## Experimental Section

### Chemicals and Apparatus

All chemicals were commercially available and used as purchased (Table 3).

**Table 3:** Sample Source, CAS Registry Number, and Initial Purity

component	chemical formula	CAS number	supplier	mass fraction (%)
3-amino-1,2,4-triazole-5-carboxylic acid	C_3_H_4_N_4_O_2_	304655-78-5	Alfa aesar, Tianjin	98
manganese chloride	MnCl_2_	7773-01-5	aladdin, Shanghai	≥99
zinc nitrate hexahydrate	Zn(NO_3_)_2_·6H_2_O	10196-18-6	aladdin, Shanghai	≥98
ethanol absolute	C_2_H_5_OH	64-17-5	aladdin, Shanghai	≥99.7
sodium hydroxide	NaOH	1310-73-2	Merck, India	96
hydrochloric acid	HCl	7647-01-0	Merck, India	37

Elemental analyses (C, H, and N) were performed on a Vario EL III analyzer. Infrared spectra were obtained from KBr pellets on a BEQ VZNDX 550 FTIR instrument within the 400–4000 cm^–1^ region. ^13^C NMR spectra were recorded on a Bruker Avance III 100 MHz spectrometer. Chemical shifts (in parts per million) were calibrated with dimethyl sulfoxide (DMSO). DSC and TG analyses were carried out on a TA Instruments NETZSCH STA 449 C simultaneous TGA at a heating rate of 10 °C·min^–1^ under hydrostatic air. *D* and *P* of detonation characteristic for EMs were estimated based on EXPLO5 v6.01.^31,32^ The density of the complex was measured by pycnometer. The heats of formation were tested by oxygen bomb calorimetry and Hess thermochemical cycle. The sensitivity to impact stimuli was determined by the fall hammer apparatus applying the standard staircase method using a 2 kg drop weight, and the results were reported in terms of height for 50% probability of explosion (h_50_). The friction sensitivity was determined on a Julius Peter’s apparatus by following the BAM method.

Diffraction data for **1** and **2** were recorded by a Bruker/Siemens Smart Apex II CCD diffractometer with graphite-monochromated MoKα radiation (λ = 0.71073 Å) at 293(2) K. Cell parameters were retrieved using SMART software and refined using SAINTPLUS^33^ for all observed reflections. Data reduction and correction for Lp and decay were performed using the SAINTPLUS software. Absorption corrections were applied using SADABS.^34^ All structures were solved by direct methods using the SHELXS program of the SHELXTL^35^ package and refined with SHELXL.^36^ Experimental details for the structural determination of the complexes are summarized in Table S1, while the selected bond lengths and bond angle data are presented in Tables S2. Hydrogen-bonding parameters are listed in Table S3.

### Synthesis of Complexes

Mn(atzc)_2_(H_2_O)_2_·2H_2_O **(1)**: compound **1** was synthesized by the solvent evaporation method. Hatzc (6.4 mg, 0.05 mmol) was dissolved in a NaOH solution (1.0 mol·L^–1^, 1.0 mL). The mixture was diluted by 5 mL of distilled water and 5 mL of EtOH, and the pH was adjusted to 6.0 with HCl solution (1.0 mol·L^–1^). MnCl_2_ (6.3 mg, 0.05 mmol) was dissolved in distilled water (5.0 mL) and then added to the above mixed solution. The reaction mixture was filtered, and the filtrate was left undisturbed at room temperature. The colorless crystals of **1** were obtained after 5 weeks (2.7 mg, yield: 43%, based on Mn^2+^). Anal. Calcd: C, 18.91; H, 3.67; and N, 29.40%. Found: C, 19.36; H, 3.16; and N, 29.89%. IR (KBr): 3523 s, 3431 s, 2360 w, 1647 s, 1636 w, 1596 s, 1522 m, 1476 m, 1404 m, 1296 s, 1103 s, 816 w, 750 m, 729 w, and 681 w. ^13^C NMR (100 MHz, DMSO): δ_c_ (ppm) 140.3, 136.5, and 120.4.

Zn(atzc)_2_(H_2_O) **(2)**: compound **2** was synthesized by the diffusion method. Hatzc (6.4 mg, 0.05 mmol) was completely dissolved in water (4 mL), which was carefully added and placed on the bottom of a test tube. Then, an ethanol solution (v/v = 1:1, 6 mL) was layered on the former. Finally, Zn(NO_3_)_2_·6H_2_O (29.8 mg, 0.1 mmol) was dissolved in EtOH (4 mL) and it was carefully layered on top. Then, it was allowed to stand at room temperature over a period of 4 weeks, whereupon colorless crystals of **2** were formed in 39% yield (2.5 mg) based on Hatzc. Anal. Calcd: C, 21.35; H, 2.37; and N, 33.19%. Found: C, 21.45; H, 2.58; and N, 33.89%. IR (KBr): 3435 s, 3335 s, 3221 w, 2962 w, 2362 w, 1653 m, 1608 w, 1531 s, 1473 m, 1387 w, 1305 s, 1120 m, 840 m, 817 m, 756 w, 729 w, and 644 w. ^13^C NMR (100 MHz, DMSO): δ_c_ (ppm) 139.6, 136.2, and 119.8.

## Supporting Information Available

Crystal data and structure refinement details, selected bond lengths and bond angles, and hydrogen bond lengths (Å) and bond angles for **1** and **2** (°); calculation parameters of *D* and *P*; TG curves of complexes **1** and **2**; DSC curves of AP; DSC curves of AP with complex **1**; and DSC curves of AP with complex **2** (PDF)
