# Supplementary material for: Thermodynamics and Catalytic Properties of Two Novel Energetic Complexes Based on 3-Amino-1,2,4-triazole-5-carboxylic Acid
Source: ACS Omega. 2022 Jan 11;7(3):3024–9. doi: 10.1021/acsomega.1c06052 (PMC8793045; doi:10.1021/acsomega.1c06052)
Supplement: Supplementary file 1 — ao1c06052_si_001.pdf [file ao1c06052_si_001.pdf]

Thermodynamics and Catalytic Properties of Two Novel Energetic Complexes Based on 3-amino-1,2,4-triazole-5-carboxylic acid

Huan Song,[a,b] Bing Li,[a,b*] Xuezhi Gao,[a,b] Fenglin Shan,[a,b] Xiaoxia Ma,[a,b] Xiaoyan Tian[a,b], Xiaoyan Chen[a,b]

[a] State Key Laboratory of High-efficiency Utilization of Coal and Green Chemical Engineering, Ningxia University.

[b] Department of Chemistry & Chemical Engineering, Ningxia University.

Index:

1. Tables ···S3

2. Graphics···S5

Table S1 Crystal data and structure refinement details of 1 and 2

Crystal	**1**	**2**
Empirical formula	$MnC_6H_{14}N_8O_8$	$ZnC_6H_8N_8O_5$
Formula weight	381.19	337.57
Crystal system	Triclinic	Monoclinic
Space group	$P\bar{1}$	$P2_1/c$
a/Å	5.2526(3)	17.094(4)
b/Å	6.5285(3)	9.507(2)
c/Å	10.6198(5)	6.7792(14)
α/º	90.605(3)	90
β/º	102.727(2)	90.001
γ/º	109.095(2)	90
V/Å^3	334.33(3)	1101.7(4)
Z	1	4
D_c (g·cm^{-3})	1.893	2.035
μ/mm^{-1}	1.051	2.270
F(000)	195	680
θ min-max/º	6.64 to 52.88	2.382 to 55.034
Goodness-of-fit on F^2	1.050	0.967
R_1/wR_2 [I＞2σ(I)]	0.0314 / 0.0797	0.0522 / 0.1339
R_1/wR_2 [all data]	0.0359 / 0.0823	0.0962 / 0.1606
Largest peak and hole/e·Å^{-3}	0.79 and -0.84	0.61 and -1.26

Table S2 Selected Bond Lengths and Bond Angles for 1 and 2

Mn(atzc)$_2$(H$_2$O)$_2$ (**1**)			
Mn(1)-N(4)^i	2.208(4)	Mn(1)-O(3)^i	2.209(3)
Mn(1)-N(4)	2.208(4)	Mn(1)-O(1)^i	2.246(3)
Mn(1)-O(3)	2.209(3)	Mn(1)-O(1)	2.246(3)
N(4)^i-Mn(1)-N(4)	180.0(2)	N(4)^i-Mn(1)-O(1)^i	75.33(11)
N(4)^i-Mn(1)-O(3)	89.31(12)	N(4)-Mn(1)-O(1)^i	104.67(11)
N(4)-Mn(1)-O(3)	90.69(13)	O(3)-Mn(1)-O(1)^i	86.58(11)
N(4)^i-Mn(1)-O(3)^i	90.69(12)	O(3)^i-Mn(1)-O(1)^i	93.42(11)
N(4)-Mn(1)-O(3)^i	89.31(13)	N(4)^i-Mn(1)-O(1)	104.67(11)
O(3)-Mn(1)-O(3)^i	179.999(1)	N(4)-Mn(1)-O(1)	75.33(11)
O(3)-Mn(1)-O(1)	93.42(11)	O(3)^i-Mn(1)-O(1)	86.58(11)
O(1)^i-Mn(1)-O(1)	180.0		
Zn(atzc)$_2$(H$_2$O) (**2**)			
Zn(1)-N(5)	1.961(8)	Zn(1)-O(5)	1.971(5)
Zn(1)-N(1)	1.986(7)	Zn(1)-O(4)	2.147(5)
Zn(1)-O(1)	2.167(5)		
N(5)-Zn(1)-O(5)	114.1(4)	N(5)-Zn(1)-N(1)	131.8(2)
O(5)-Zn(1)-N(1)	114.0(4)	N(5)-Zn(1)-O(4)	79.1(2)

O(5)-Zn(1)-O(4)	96.9(2)	N(1)-Zn(1)-O(4)	97.4(2)
N(5)-Zn(1)-O(1)	95.2(2)	O(5)-Zn(1)-O(1)	93.5(2)
N(1)-Zn(1)-O(1)	79.7(2)	O(4)-Zn(1)-O(1)	169.5(2)

Symmetry transformations used to generate equivalent atoms: i 1 -x+1,-y+1,-z+1

Table S3 Hydrogen Bond Lengths (Å) and Bond Angles (°) for 1 and 2

D–H···A	d(D–H)	d(H···A)	d(D···A)	∠(DHA)
$Mn(atzc)_2(H_2O)_2$ (**1**)				
N(1)–H(1A)···O(3)	0.86	2.14	2.9240	152
N(1)–H(1B)···N(3)	0.86	2.17	2.9074	143
N(2)–H(2)···O(2)	0.86	2.03	2.8869	172
O(3)–H(3A)···O(1)	0.87	2.11	2.7388	129
O(3)–H(3B)···O(2)	0.87	1.89	2.7536	171
$Zn(atzc)_2(H_2O)$ (**2**)				
N(8)–H(8A)···N(7)^{i}	0.86	2.12	2.946(10)	159.7
N(8)–H(8B)···O(1)	0.86	2.40	3.130(9)	143.7
N(7)–H(7)···N(8)^{ii}	0.86	2.12	2.946(10)	161.6
N(4)–H(4B)···O(5)^{iii}	0.86	2.57	3.271(11)	139.2
N(4)–H(4B)···O(4)	0.86	2.63	3.204(9)	125.3
N(4)–H(4A)···N(3)^{iv}	0.86	2.24	3.034(10)	153.5
N(3)–H(3)···N(4)^{v}	0.86	2.20	3.034(10)	163.0
O(5)–H(5B)···O(2)^{vi}	0.89	2.03	2.797(10)	143.4
O(5)–H(5A)···O(4)^{vii}	0.89	2.17	3.042(7)	168.0
O(5)–H(5A)···O(3)^{vii}	0.89	2.09	2.732(10)	128.3

Symmetry transformations used to generate equivalent atoms: i -x,y-1/2,-z+1/2; ii -x,y+1/2,-z+1/2; iii x,-y+1/2,z-1/2; iv -x+1,y+1/2,-z+1/2; v -x+1,y-1/2,-z+1/2; vi x,-y-1/2,z+1/2; vii x,-y+1/2,z+1/2

1 Energetic Properties for complexes 1 and 2.

The heats of formation for complexes **1** and **2** were calculated with oxygen bomb calorimetry and Hess thermochemical cycle. The calculated $\Delta_c H$ values of **1** and **2** are -13.02 and -11.98 kJ·g^{-1}, respectively. The enthalpies of formation ($\Delta_f H^o$) of the two compounds were calculated by the Hess thermochemical cycle, as shown in equations 1 and 2:

$$\Delta_f H^o[\mathbf{1}, s]=\Delta_f H^o[MnO_2, s]+6\Delta_f H^o[CO_2, g]+7\Delta_f H^o[H_2O, l]-\Delta c H^o[\mathbf{1}, s] \quad (1)$$

$$\Delta_f H^o[\mathbf{2}, s]=\Delta_f H^o[ZnO, s]+6\Delta_f H^o[CO_2, g]+4\Delta_f H^o[H_2O, l]-\Delta c H^o[\mathbf{1}, s] \quad (2)$$

where $\Delta_f H^o$ (MnO_2, s)= −520.00 kJ·mol^{-1}, $\Delta_f H^o$ (ZnO, s) = −348.00 kJ·mol^{-1}, $\Delta_f H^o$ (CO_2, g) = −393.51 kJ·mol^{-1},, and $\Delta_f H^o$ (H_2O, l) = −285.83 kJ·mol^{-1}. The $\Delta_f H^o$ values for 1 and 2 were 0.21MJ·kg^{-1}, 0.56 MJ·kg^{-1}, respectively.

Detonation velocity (D) and detonation pressure (P) are the particularly vital targets to illustrate the detonation characteristic of energetic materials. For the studying of metal containing energetic materials, D and P were usually calculated with the EXPLO5 code. The calculation results are shown in the Table S4.

Table S4 Calculation parameters of detonation velocity (D) and detonation pressure (P)

complex	ρ (g·cm^{-3})	N (mol·g^{-1})	M (g·mol^{-1})	D (km·s^{-1})	P (GPa)
**1**	1.853	0.024	22.320	10.4	48.7
**2**	1.998	0.024	23.000	10.2	48.6

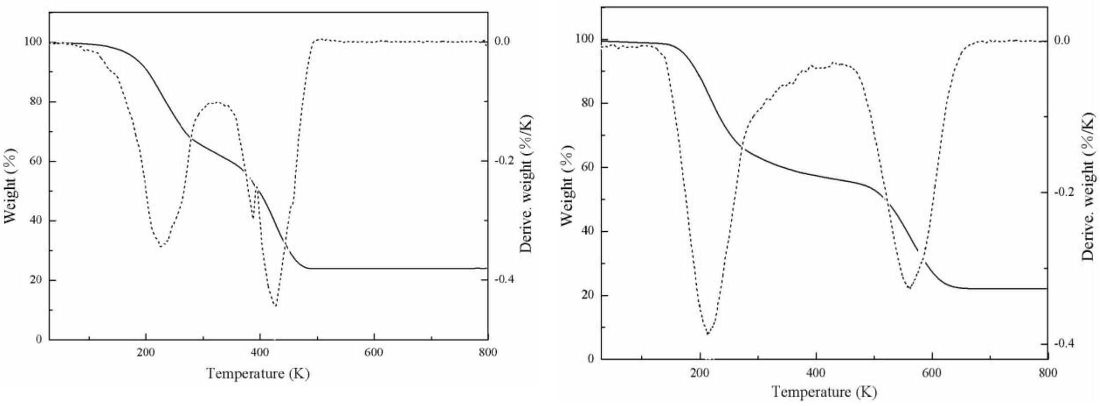

Figure S1. The TG curves of complexes 1 (left), 2 (right) with the heating rate of 10 ℃·min^{-1}.

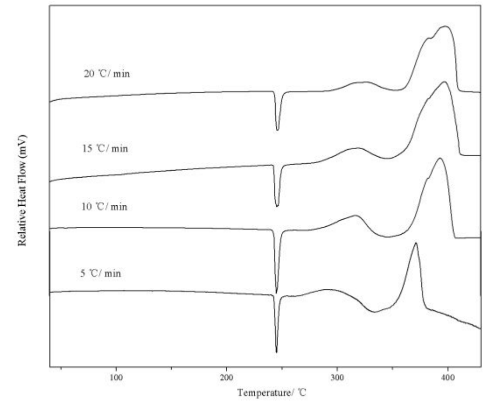

Figure S2. DSC curves of AP at various heating rates.

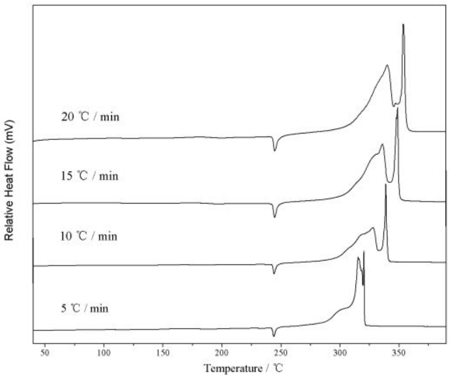

Figure S3. DSC curves of AP with complex 1 at various heating rates.

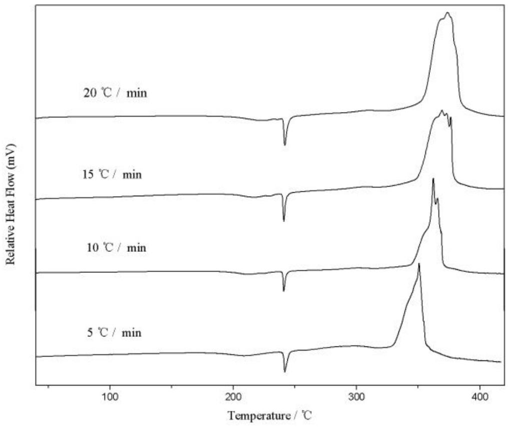

Figure S4. DSC curves of AP with complex 2 at various heating rates.
